# A Bird’s-Eye View of Cell Sources for Cell-Based Therapies in Blood Cancers

**DOI:** 10.3390/cancers12051333

**Published:** 2020-05-23

**Authors:** Benjamin Motais, Sandra Charvátová, Matouš Hrdinka, Michal Šimíček, Tomáš Jelínek, Tereza Ševčíková, Zdeněk Kořístek, Roman Hájek, Juli R. Bagó

**Affiliations:** 1 Faculty of Medicine, University of Ostrava, 703 00 Ostrava, Czech Republic; benjamin.motais@fno.cz (B.M.); sandra.charvatova@fno.cz (S.C.); matous.hrdinka@fno.cz (M.H.); michal.simicek@fno.cz (M.Š.); tomas.jelinek@fno.cz (T.J.); tereza.sevcikova@fno.cz (T.Š.); zdenek.koristek@fno.cz (Z.K.); roman.hajek@fno.cz (R.H.); 2 Faculty of Science, University of Ostrava, 701 03 Ostrava, Czech Republic; 3 Department of Haematooncology, University Hospital Ostrava, 708 52 Ostrava, Czech Republic

**Keywords:** cell-based therapy, blood cancer, CAR-T cells, NK cells, dendritic cells, platelets, macrophages, iPSC, stem cells

## Abstract

Hematological malignancies comprise over a hundred different types of cancers and account for around 6.5% of all cancers. Despite the significant improvements in diagnosis and treatment, many of those cancers remain incurable. In recent years, cancer cell-based therapy has become a promising approach to treat those incurable hematological malignancies with striking results in different clinical trials. The most investigated, and the one that has advanced the most, is the cell-based therapy with T lymphocytes modified with chimeric antigen receptors. Those promising initial results prepared the ground to explore other cell-based therapies to treat patients with blood cancer. In this review, we want to provide an overview of the different types of cell-based therapies in blood cancer, describing them according to the cell source.

## 1. Introduction

**Figure 1:**
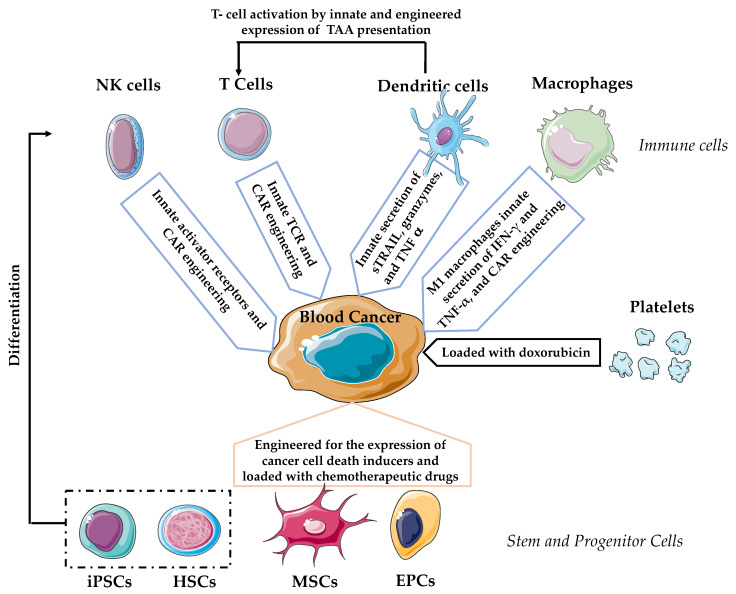
Diagram depicting the different cell-based therapies to treat hematological malignancies with the mode of action in each case. NK: natural killer, iPSCs: induced pluripotent stem cells, HSCs: hematopoietic stem cells, MSCs: mesenchymal stem cells, and EPCs: endothelial progenitor cells.

Hematologic malignancies are the fourth-most common type of cancer, with a higher incidence in older adults [1]. The number of cases is expected to rise over the years because of the increase in life expectancy [2]. Traditionally, the treatment regimens have included chemo and radiotherapy and stem cell transplantation. In the past decade, substantial improvements in patients’ outcomes have been achieved through advances in diagnosis and treatment. Some of those new therapeutic approaches include the administration of monoclonal antibodies [3,4,5,6,7] and immunomodulatory drugs [8]. Unfortunately, some types of blood cancers remain incurable. In a rapid-fire series of breakthroughs in recent years, cancer cell-based therapy is flourishing as a novel and promising approach to combat otherwise incurable hematologic malignancies [9,10,11]. The cancer patients undergoing such cell-based therapies are administrated a “living drug” in the form of modified or unmodified living cells from the patient or a suitable donor, which are able to specifically recognize and destroy malignant cells. In practical clinical development, cell-based therapies with T lymphocytes modified with chimeric antigen receptors (CARs) have advanced the most, with striking results in the treatment of different types of hematological cancers [12,13]. In this review, we aim to give an overview of the different types of cancer cell-based therapies to treat hematological malignancies (Figure 1), organizing them according to the cell type and source used as a therapeutic vehicle and highlighting their main benefits and remaining challenges. 

## 2. T Lymphocytes

T cells are apparent candidates for cancer cell-based immunotherapy due to their inherent activity against cancer. T lymphocytes kill tumor cells upon T cell receptor (TCR) recognition of cancer-specific antigens presented by the major histocompatibility complex (MHC). When activated by an antigen, the intracellular domains of CD3ζ in the TCR complex become phosphorylated on their immunoreceptor tyrosine-based activation motifs and triggers a signaling cascade resulting in the expression of transcription factors such as AP-1, NF-κB, or NFAT. In this activated state, the T cells express and release cytolytic enzymes such as granzymes and perforins, thus inducing apoptosis in the target tumor cells. Harnessing the natural recognition of cancer antigens, immunotherapies based on activated T cell transplantation have shown positive results in the past [14].

More recently, the introduction of novel T cell therapies based on CAR-T technology has genuinely revolutionized the battle against cancer, particularly in hematological malignancies. This technology was first described by Gross and colleagues at the Weizmann Institute of Science in Israel in the 1980s [15]. With the CAR approach, immune T cells are armed with artificial receptors that directly recognize specific epitopes present on the surface of tumors cells, bypassing the need for the classical antigen presentation process. The CARs typically consist of an extracellular antibody-derived single-chain variable fragment (scFv) that recognizes specific tumor-related antigens, a single transmembrane domain, and an intracellular signal domain responsible for triggering the cellular immune responses [16]. Over the years, several generations of CARs have been engineered in order to improve the efficacy of CAR-T cells. The first CAR generation consists of a single intracellular signaling domain of CD3ζ derived from the natural TCR complex. With the intention to boost the T cell response after antigen recognition, an addition of one or two costimulatory domains, such as signaling domains CD28 [17] and 4-1BB [18], were added to the first generation of CAR, leading to the second (one additional domain) and third (two additional domains) generations of CARs. The fourth generation of CARs, also referred to as “TRUCKs” (T cells Redirected for Universal Cytokine-mediated Killing), enables the expression of immune-stimulatory cytokines, such as IL-2, to enhance CAR-T cell persistence, expansion, and resistance to immunosuppression [19]. Over the years, rapid advances in genetic engineering have allowed the creation of more sophisticated CAR-T designs to be tested. For example, dual-target CAR-T cells have been developed to provide T-cells with two different scFvs, increasing the killing efficiency and reducing tumor escape in heterogeneous tumors [20]. Furthermore, the efforts to produce more flexible and modulated CAR-T cells in terms of antigen specificity and activity has resulted in the generation of SUPRA (Split Universal Programmable) CARs. The intracellular parts of SUPRA CARs are based on traditional signaling domains, but instead of an extracellular antigen-binding scFv, they display a high-affinity binding domain of leucine zippers [21]. The fully functional CAR is then formed when leucine zippers in the cell membrane match other leucine zippers attached to a specific soluble scFv. This elegant solution simultaneously addressed both the regulation of CAR specificity (targeting various antigens) and activity (creating an active CAR on demand). Other varieties of switchable CARs have been proposed, such as the one with the biotin-avidin system [22]. Those and other switchable designs are flexible systems, allowing an easy and quick reprogram of the CAR, expanding the possible applications and targets and reducing the cost of CAR-T manufacturing. To sum up, T cells can be armed with many viable CAR options, all of which possess unique advantages and disadvantages that will be further defined in the following preclinical and clinical studies.

In most of the cases, before treatment with CAR-T cells, patients with hematological cancers receive lymphodepleting chemotherapy, which permits an appropriate immune environment for the CAR-T cell transfer, improving their in vivo function, progression, and persistence [23].

As of 2019, according to the CARGlobalTrials online database [24], there are a total of 353 CAR-T cell clinical trials involving 16,232 patients with hematological cancers, with 52% trials in phase I. So far, two anti-CD19 CAR-T products, Kymriah (Novartis) and Yescarta (Gilead), have received marketing authorization from the US Food and Drug Administration and the European Medicine Agency. Kymriah is indicated for the treatment of young adults and pediatric patients with refractory or relapsed acute lymphoblastic leukemia (ALL), whereas Yescarta is for the treatment of large B cell lymphoma in adult patients. Kymriah was the very first CAR-T product approved, after a successful phase I trial with the second generation of CAR (with 4-1BB as a costimulatory domain) targeting CD19. Out of the 30 patients tested with B- and T-cell ALL, 19 had a sustained complete remission and successfully recovered from cytokine release syndromes triggered by therapy [25]. A second-phase clinical trial is currently being performed in 101 patients with non-Hodgkin’s lymphoma (NHL), and thus far, 54% of patients are showing a complete remission [26]. Many other clinical trials are ongoing, most of them against CD19 and others targeting BCMA (B-cell maturation antigen) [27], CD20 [28], CD22 [29], CD30 [30], or LeY [31].

The engineering of T cells to express the CAR transgene is mainly accomplished using lentiviral [32] and retroviral vectors [33]. However, due to safety concerns inherent to lentiviral transduction [34], nonviral delivery vectors are being intensively studied. Nonviral delivery systems, such as the transposition method with Sleeping Beauty [35] and PiggyBac [36] or based on CRISPR-Cas9 technology [37], have already proved to achieve a stable integration and expression of CAR in T cells.

New studies point out the importance of the T cell ratio between cytotoxic CD8+ T cells and helper CD4+ T cells [38] and a subset selection of those T cells depending on their state of differentiation [39,40,41] to achieve efficacy in CAR-T cell immunotherapy. Recent clinical trials, such as JCAR017 from JUNO Therapeutics [42], are delivering a particular ratio of CD4+ and CD8+ CAR-T cells to reduce T cell malfunction and apoptosis.

While CAR-T cells are a huge step towards a cancer cure, they are not without drawbacks. One major concern is the manufacturing costs of autologous T cells for each patient, resulting in expensive treatment. The overall costs could be reduced by using an allogeneic T cell source, which is, however, burdened with life-threatening complications such as Graft-versus-host-disease (GvHD), as well as graft rejection by the host immune system. GvHD occurs when infused T cells from a donor get activated via TCR after recognizing the human leukocyte antigen (HLA) mismatch from the patient, resulting in donor T cells attacking the healthy patient’s tissues. Graft rejection is caused by the patient’s immune system, which attacks donor T cells after recognizing the HLA mismatch. To circumvent these problems and open the way to the use of allogenic T cells, new lines of research aimed at the generation of universal, off-the-shelf T cells with missing TCR and HLA molecules to avoid GvHD and graft rejection, respectively [43]. Another shortcoming of CAR-T cell therapy is the toxicity linked to the application of CAR-T cells. The side effects observed are neurologic toxicity, cytokine release syndrome, “on-target/off-tumor” recognition, and anaphylaxis [44]. These symptoms usually appear in clinical trials at different grades and may cause minor-to-severe side effects. To mitigate those undesired secondary effects, researchers have designed different strategies to eliminate or control CAR-T cell activity in case of severe toxicity [45]. These strategies include the expression of inducible suicide genes such as the Herpes simplex virus thymidine kinase [46] and caspase 9 [47], the expression of CD20 [48] and the epidermal growth factor receptor [49] to mediate a suicide switch with antibodies, CAR activation that requires the recognition of two different tumor antigens [50], the expression of a synthetic Notch that regulates the transcription of the CAR [51], the expression of immune inhibitory receptors [52], and on-switch CARs activated by a small molecule [53]. The most employed so far in clinical trials to treat hematological malignancies has been the suicide switch with antibodies, with a significant impact on the control of the undesired side effects. The major downside of that approach is the possible injury to healthy tissues that express the same antigen as the one recognized by the antibody. In consequence, this may restrict its future development, facilitating its substitution by the other approaches, where the damage to healthy cells is avoided.

## 3. Natural Killer (NK) Cells

Cancer immunotherapy based on NK cells has gradually risen over the past few years as an attractive and promising alternative to CAR-T cell therapy. The unique biological characteristics of NK cells allow us to circumvent two main limitations observed in the CAR-T cells. First, the tumor cells escape from T cell surveillance. The full immune response against cancer cells exerted by T cells relies on MHC-I recognition. Unfortunately, tumors have a propensity to downregulate MHC-I, leading to the escape of T cell antitumor actions [54]. In contrast to T cells, NK cells can exhibit a direct cytotoxic effect against tumor tissues lacking the expression of MHC-I [55]. NK cells express different activating and inhibitory receptors that, upon binding to specific ligands, govern the cytotoxic response. Some examples of activating receptors expressed by NK cells are NKp46, NK1.1, NKG2D, CD16, and CD244. NK cell inhibitory receptors fall into two groups: the monomeric type I glycoproteins of the immunoglobulin superfamily and the type II glycoproteins with a C-type lectin-like scaffold. Examples of type I are killer cell immunoglobulin-like receptors and leukocyte immunoglobulin-like receptors that recognize specific MHC-I molecules. Second, as the function of NK cells relies on the balance of signals from inhibitory and activating receptors that recognize a specific pattern of ligands in healthy cells and diseased ones, the infusion of allogeneic NK cells is safe and does not cause unwanted and deleterious GvHD [56], thus laying the foundation for the development of another universal, off-the-shelf cancer cell-based immunotherapy.

Different cell sources have been proposed for NK cell-based cancer therapy. Interestingly, one approach utilizes established NK cell lines, such as the NK-92 cell line, obtained from a patient with clonal NK cell lymphoma. The main advantage is that the NK-92 cell line can be very easily expanded in vitro and retains high antitumor activity [57]. This high activity is due to a consequence of their unique expression profile of receptors, with few inhibitory receptors and a relatively large amount of activating receptors [58]. A significant disadvantage, however, is the need to irradiate the therapeutic cells before the infusion to completely abrogate their proliferation, reducing, in consequence, the in vivo persistence of the effector cells.

Traditionally, NK cells are obtained from donor peripheral blood mononuclear cells [59] and umbilical cord blood [60] but, also, from stem cell sources, such as umbilical cord blood hematopoietic stem cells [61] and human pluripotent stem cells [62]. Each cell source has its inherent advantages and disadvantages, and more preclinical studies will be needed to untangle what the best source of NK cells is.

Similar to T cell-based therapies, NK cells have been armed with different CARs to boost their antitumor efficacy. The CAR structure is analogous to CARs used in T cells. In some cases, however, the signaling domain is slightly modified to be more adaptive to the characteristics of NK cells [63]. Several preclinical studies have already demonstrated the feasibility and benefits of CAR-NK technology. For example, the NK-92 cell line expressing an anti-CD138 CAR [64] showed a significantly enhanced cytotoxicity when compared to control NK-92 cells. In vivo experiments highlighted a significant reduction of the multiple myeloma (MM) tumor volume and increased the survival rate in xenograft mice models treated with anti-CD138 CAR NK-92 cells.

In 2019, the CARGlobalTrials database registered 11 clinical trials utilizing CAR-NK cells, with 174 patients enrolled. To date, only two studies have disclosed their results. The first disclosed phase I clinical trial was performed in three patients with acute myeloid leukemia (AML) with an infusion of NK-92 cells targeting CD33+ tumor cells [65]. The transplanted NK 92 cells did not produce any adverse effects on patients but also did not demonstrate any clinical efficacy. The second disclosed phase I/IIa clinical trial was conducted at the University of Texas MD Anderson Cancer Center and used CD19 CAR-NK cells derived from cord blood to treat 11 patients with relapsed or refractory NHL and chronic lymphocytic leukemia (CLL) [66]. In this case, the study revealed a favorable clinical outcome, with a 73% response rate without significant toxicities.

Overall, CAR-NK cells have demonstrated a great potential to overcome CAR-T cell limitations. However, the CAR-NK cell approach is a recent concept and, therefore, needs to be further improved to surpass its challenges. One of them is the resilience of the NK cells to be genetically engineered by viral and conventional nonviral gene-editing techniques [67]. The use of the endogenous baboon virus was recently found to significantly increase the transduction efficiency of NK cells as compared to VSV-G or RD114 pseudotyped lentiviruses [68]. Electroporation or use of cationic polymers on NK cells have also shown some limitations, even though optimized protocols have been published [69,70].

## 4. Dendritic Cells (DCs)

In 1973, DCs were discovered in mice by Ralph M. Steinman [71], who also elucidated their essential role in adaptive immunity. For his discoveries, he was awarded the Nobel Prize in Physiology or Medicine in 2011. DCs are part of the innate immune system and play a key role in antigen presentation. DCs have no direct killing activity against cancer cells but can present tumor-associated antigens (TAAs) to naïve T cells, leading to their activation. They can also activate other immune cells, such as NK cells. Therefore, the infusion of dendritic cells, called DC vaccines, has been proposed as a way of immune system reactivation in cancer patients by overcoming endogenous DC malfunctions [72,73] and, in turn, enhancing T cell responses against tumor cells.

Several clinical trials employing different approaches have been performed with DCs to treat patients with hematological cancers. In one approach, DCs were loaded in vitro with specific TAAs, applying diverse methods such as incubation with peptides derived from tumors [74]; tumor apoptotic bodies [75]; or whole tumor cells (lysed, heat-killed, or irradiated) [76]. Other teams directly engineered the DCs to express specific antigens [77] or even used DCs derived from leukemia patients naturally displaying TAAs on their surface [78]. Another approach selected a specific subset of cytotoxic DCs (killer DCs), which show the particularity to secrete cytotoxic molecules such as soluble TNF-related apoptosis-inducing ligand (sTRAIL), granzymes, or TNF-α [79]. The majority of these clinical trials reported safety, with mild side effects and an overall survival advantage.

Since DCs represent less than 1% of peripheral blood mononuclear cells, obtaining therapeutically meaningful numbers for vaccination purposes is one of the main problems. This problem can be bypassed by an allogeneic source of DCs, as they do not cause GvHD, or, alternatively, by the ex-vivo differentiation of CD14+ monocytes to DCs [80].

Overall, DC vaccines hold potential in blood cancer cell therapy, as the preclinical and clinical results are promising. Nevertheless, this field is still in its infancy, and more studies need to be carried out to improve the efficacy and elucidate the exact working mechanisms.

## 5. Macrophages

Macrophages are a type of specialized innate immune cells implicated in the detection, phagocytosis, and elimination of cellular debris, pathogens, and cancer cells. They are also involved in antigen presentation to T cells.

The commencement of macrophage-based therapy in cancer can be traced back to the work of Dr. Isaiah Fidler, who used macrophages previously cultured in vitro with conditioned media from B16 tumor cells (mouse melanoma cells) and sensitized lymphocytes. Such stimulated macrophages were then infused in mice with subcutaneous B16 tumors and achieved a significant reduction in pulmonary metastases [81]. From 1987 to 2010, autologous macrophages were employed in clinical trials to treat different types of solid tumors, with nonserious side effects in various dose-escalation regimens but with moderate therapeutic efficacy and no long-term remissions [82]. Monocytes from peripheral blood were the cell source in most of the cases. Once isolated, they were expanded ex vivo and differentiated to the cytotoxic phenotype (M1 macrophages) with 100–1000 U/mL human interferon-gamma (IFN-γ) before infusion [83].

Today, we have better understanding of the biodistribution and mechanism of action of macrophages. Two main reasons could be used to explain its failure in clinical trials, the tissue distribution of infused macrophages being the first. The tissue distribution of labeled macrophages with 111indium after intravenous infusion in patients with renal carcinoma shows infiltration in the lungs, liver, and spleen but with a lack of trafficking into the tumor [84]. The second is the tumor microenvironment’s capacity to polarize M1 macrophages towards an M2 macrophage’s phenotype, which is related to wound healing and tissue repair, thus boosting the tumor’s malignancy [85,86].

Recent preclinical studies have been focused on surpassing those pitfalls and increasing the macrophages’ efficacy as effector cells by genetic modifications [87,88,89] or by loading them with anticancer drugs or nanoparticles for photothermal therapies [90].

To date, no clinical trials with macrophages have been performed in hematological malignancies, and only a few preclinical studies have been reported. One of those preclinical approaches engineered macrophages to express a new type of CAR that activates their phagocytic mechanism after the recognition of CD19 in Burkitt’s lymphoma cell line Raji [91]. This novel CAR has an extracellular scFv that recognizes the antigen CD19 and an intracellular domain composed of Megf10 or FcRɣ capable of triggering phagocytosis after antigen recognition. The researchers observed that the engineered macrophages cocultured in vitro with the Raji cell line were able to engulf six cancer cells per 100 macrophages, reducing the number of cancer cells by 40%.

The majority of the recent striking trials in blood cancer cell-based therapy have been performed using the immune cells described above and summarized in Table 1.

## 6. Platelets

Platelets, or thrombocytes, are non-nucleated cell fragments. Their primary function is the formation of clots after blood vessel injury to stop bleeding and to maintain vascular integrity. Harnessing their capacity to adhere to tumor cells [97], platelets have been studied as a vehicle to deliver therapeutic drugs to cancer cells [98]. In a recent preclinical study, platelets were loaded with doxorubicin to deliver the cytotoxic agent to lymphoma cells [99]. The study proved the localized delivery of the drug to the tumor, increasing the therapeutic efficacy and reducing the cardiotoxicity associated with the chemotherapeutic agent.

Platelets are a promising drug delivery vehicle for cancer treatment because of their abundance [100] and their capacity to adhere to cancer cells. However, platelets can be a double-edged sword, as they also play an essential role in the stimulation of tumor dissemination and proliferation by acting on tumor angiogenesis [101], metastasis [102], growth [103], and, in chemotherapy, resistance [104].

## 7. Stem Cells and Progenitor Cells

Stem cells (SCs) are a particular type of cells with a high self-renewal capacity and exceptional ability to differentiate into many specialized cell types. Depending on their differentiation capacity, they can be classified as pluripotent or multipotent. Among the pluripotent SCs, we have the embryonic SCs and the induced pluripotent SCs, with the ability to differentiate into all three germ layers: mesoderm, endoderm, and ectoderm. In the group of multipotent SCs, we have the adult SCs that can be found throughout the body, with a more limited capacity to differentiate to diverse cell types. Examples of adult SCs are the hematopoietic SCs, the mesenchymal SCs, and the endothelial progenitor cells. They play the central role in the homeostasis and regeneration of all body tissues. SCs exhibit inherent tropism for cancer cells, which makes them an ideal therapeutic vehicle in anticancer therapy [105,106] (Table 2).

### 7.1. Hematopoietic Stem Cells (HSCs)

HSCs are adult multipotent stem cells responsible for generating all the blood cell types in the bone marrow by a process called hematopoiesis. The use of HSCs for cancer treatment is not new. The first successful HSC transplantation was accomplished by Dr. E. Donnall Thomas in 1957 in a patient with acute leukemia [112]. He received the Nobel Prize in Physiology or Medicine for this groundbreaking work. Nowadays, HSC transplantation is an established treatment for leukemia and other hematological malignancies [113,114], to overcome hematopoietic failure during the high doses of chemotherapy. HSC transplantation can be autologous, allogeneic, or syngeneic, and the cell source can be peripheral blood, bone marrow, or umbilical cord blood [115]. The selection of the source depends on the transplantation indication [116], as well as donor availability. In the allogeneic transplantation of HSCs, the immune cells in the graft can exert an immune response against residual malignant cells, in a process known as the graft versus tumor effect (GvT). An interesting recent approach used the ex vivo gene modification of those HSCs to boost the GvT by the expression of CARs or by the prearranging of the TCR. Thus, the modified HSC population transplanted into the patient can become a long-lasting source of T or NK cells engineered to recognize specific tumor antigens [117]. A recent preclinical study in 2019 has engineered HSC to express NY-ESO-1 TCR. They show how these cells can differentiate into all blood lineages, along with persistence and safety after transplantation to myelo-depleted HLA-A2/K^b^ mice. This preclinical study was performed to authorize an investigational new drug application for a clinical trial that is currently being conducted at the University of California, Los Angeles, to treat patients in stage IV or locally advanced unresectable cancers [118]. In the future, this approach could be applied in patients with a hematological cancer, as the NY-ESO-1 TCR can recognize the immunogenic cancer-testis antigen NY-ESO-1 expressed in more than 60% of advanced MM [119].

Whether these new approaches will lead to an increase in efficacy compared to the traditional HSC transplantation remains to be proven in clinical trials. Regardless, the use of genetically modified HSCs is an appealing approach, as it can provide a life-long source of effector immune cells engineered against the specific antigen and can continuously replenish exhausted immune cells.

### 7.2. Mesenchymal Stem Cells (MSCs)

MSCs are multipotent stromal cells that can differentiate to many cell types, such as osteoblasts, chondrocytes, and myocytes [120]. As a consequence of this unique capacity, MSCs have been extensively studied for tissue regeneration purposes [121,122,123,124]. More recently, MSCs have been proposed as a vehicle for targeted tumor therapy because of their tumor-tropic properties and relative resistance to chemotherapeutic drugs [125,126]. Tumor cells and tumor microenvironments secrete chemoattractant factors that induce MSC homing [127]. For instance, the expression of CCL25 by MM cells has been proved to attract MSCs after interaction with their receptor CCR9 [128]. The migration capacity of MSCs has also been studied in the context of radiation therapy, increasing their interest as a therapeutic vehicle for cancer treatment. Indeed, Klopp et al. [129] showed that MSCs have a better migration towards irradiated mouse mammary tumor cells (4T1) compared to nonirradiated ones. The migration enhancement after the irradiation of tumor cells was a consequence of the upregulation of cytokines involved in MSC migration, such as VEGF, PDGF-BB, TGF-β, and SDF-1. Besides, they observed that MSCs in the presence of irradiated tumor cells upregulated the expression of the chemokine receptor CCR2. Harnessing the tumor-tropic capacity of MSCs, Li et al. [130] and Ciavarella et al. [109] demonstrated the killing capacity of MSC towards leukemia and myeloma cells after engineering them for the expression of human interferon-gamma (IFN-γ) and TRAIL, respectively. Bonomi et al. [131] and Pessina et al. [108] took advantage of the resistance to chemotherapeutic drugs and tumor-tropic properties of MSCs and loaded them with paclitaxel (10,000 ng/mL) to suppress proliferation of the human MM cell line RPMI8226 and the leukemia cell line MOLT-4 in different preclinical studies. This capacity to absorb and release paclitaxel in tumors has also been proved in other chemotherapeutic drugs, such as doxorubicin and gentamicin, with an in vitro inhibition of cell growth on tongue squamous cell carcinoma [132].

Other assets of the MSCs are their relatively easy isolation, expansion, and genetic modification [133]. Besides, they have many possible tissue sources, such as bone marrow; peripheral blood; adipose tissue; or neonatal birth-associated tissues such as the cord blood, umbilical cord, or placenta [134]. The function of MSCs in hematological cancers is less-known than in solid tumors, but they share the same controversy. Probably because of the heterogeneity in the MSC population, there are studies that claim their antitumor properties [135]; others show the opposite due to evidence that they favor tumor growth [136]. The antitumor effect of MSCs in blood cancers has been reported primarily as a consequence of the suppression in the proliferation of malignant cells by the cell cycle arrest [137,138,139]. On the other hand, the MSCs have been stated to promote different hematological malignancies by activating metastasis/recurrence [140], suppressing apoptosis [141,142], involvement in the immunomodulation of cancer cells [143,144], supporting tumor vasculature [145], and inducing drug resistance [146,147]. More conclusive and standardized studies need to be performed in this matter before MSCs can be considered as an efficient therapeutic vehicle in hematological malignancies [148].

### 7.3. Endothelial Progenitor Cells (EPCs)

The definition of EPCs is still problematic due to their lack of specific markers. At the moment, they are characterized according to their capacity to differentiate to mature endothelial cells, to proliferate and migrate, and by functional parameters such as the ability to form vessels in vivo and tubular-like structures in vitro [149]. EPCs can be obtained from peripheral blood, bone marrow, cord blood, and other tissues such as adipose tissue [150].

Due to their tumor-homing properties, EPCs have been studied as a vehicle to deliver different therapeutic agents to tumors by the transgene expression of antiangiogenic agents, suicide genes, immune stimulators, or even employed as a virus and nanoparticle carrier to increase the primary therapeutic efficacy [151]. So far, no clinical trials with EPCs as a cancer therapeutic vehicle have been performed. The major hindrances in the translation of EPCs into the clinics are the absence of standardization in isolation and expansion, low numbers of EPCs after isolation [152], the immunogenicity in allogeneic sources, and their inherent protumor proliferation properties [153].

### 7.4. Induced Pluripotent Stem Cells (iPSCs)

iPSCs are derived from somatic cells by the expression of key transcription factors (Myc, Oct3/4, Sox2, and Klf4). The expression of those transcription factors reprograms the somatic cells into an embryonic-like pluripotent nature that permits the generation of an unlimited source of a specific cell type after the induction of differentiation. This revolutionary discovery was made by Takahashi and Yamanaka in 2006 [154], who were subsequently awarded the Nobel Prize in 2012. iPSCs derived from fibroblasts have been successfully differentiated into functional tumor-targeting T and NK cells. In 2009, Lei et al. [155] were the first to state that the differentiation of iPSCs to T lymphocytes is possible through the coculture of iPSCs (iPS-MEF-Ng-20D-17 cell line) with OP9 stromal cells expressing the Notch ligand Delta-like 1. Transfer of these cells into *Rag*-deficient mice restored T cell pools and generated mature T lymphocytes. A year later, Watari et al. [156] successfully obtained fully functional natural killer T cells (NKT) from iPSCs derived from murine embryonic fibroblasts. These NKT cells derived from iPSCs were able to suppress EG7 (murine T cell lymphoma) tumor growth in vivo in *α18–/–* mice. In 2013, Knorr et al. [110] were able to obtain a large number of cytotoxic NK cells from iPSCs derived from hematopoietic progenitor cells in a feeder-free system. In the same year, Themeli et al. [111] successfully combined iPSCs and CAR technologies to produce human T cells that target CD19 in malignant B cells. A recent publication by Li Zhang et al. [157] obtained functional macrophages of iPSC derived from peripheral blood mononuclear cells. These macrophages were engineered for the expression of CD19 CAR to trigger phagocytosis after tumor antigen recognition in leukemia cell lines Nalm6 and K562. Though some anticancer activity was observed in mouse models of leukemia, the results are not conclusive as a consequence of high variability, requiring further development.

At the moment, the trend is leading towards the development of T and NK cells derived from iPSCs that can be delivered off-the-shelf, simplifying the manufacturing process and reducing the overall costs compared to traditional approaches using autologous cells. A significant drawback in cells derived from iPSCs is the potential risk of teratoma formation due to the activation of pluripotency genes [158]. In the future, inducible CRISPR-Cas9 technology could be used to permanently turn off or even delete these genes.

## 8. Conclusions

**Figure 2:**
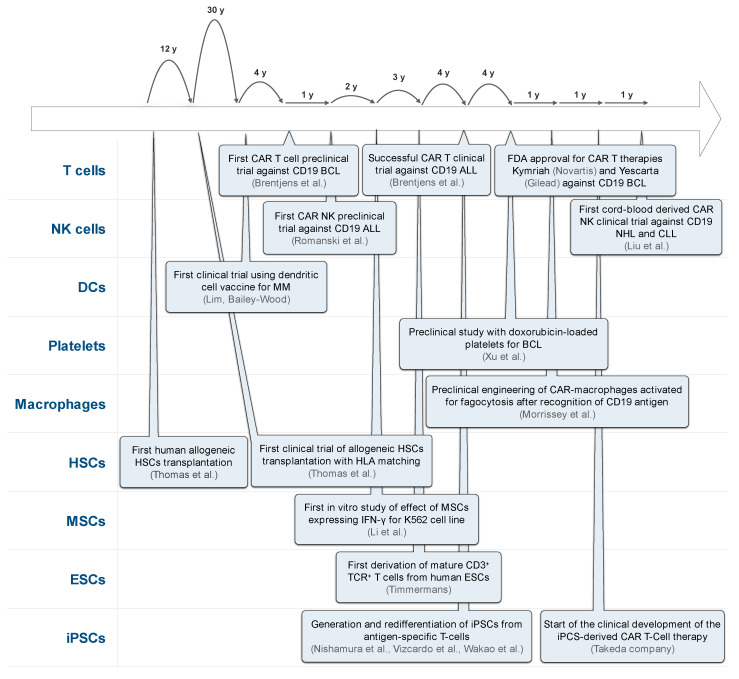
Timeline of cancer cell-based therapy milestones in the past 70 years in blood cancer treatments. ALL: acute lymphoblastic leukemia, BCL: B cell lymphoma, CLL: chronic lymphocytic leukemia, ESCs: embryonic stem cells, NHL: Non-Hodgkin’s lymphoma, MM: multiple myeloma.

As a consequence of the promising results obtained in the recent clinical trials, cancer cell-based therapy is flourishing as a new pillar in cancer treatment and is likely to become the cornerstone in future blood cancer treatments. However, for the time being, cancer cell-based therapy is a fledgling, and therefore, there is still a long road ahead **(**Figure 2). To consolidate this novel approach will require more basic and translational research to solve roadblocks such as effector toxicity, persistence, homing, tumor escape, and universal access. A better understanding of the different cell sources available may help to improve the future cell-based therapeutic approaches to treat hematological malignancies by selecting the proper cell type to increase the efficacy and to reduce toxicity and the cost of production. In saying that, a universal effector cell source for different cancers may not exist, and perhaps, it will be required to identify the best cell source for each type of cancer or the best combination of different effector cells to tackle specific cancer cell types.

## Figures and Tables

**Table 1:** Selected cell-based clinical trials with immune cells to treat hematological malignancies featuring key aspects.

Year	Cell Source	Target	Engineering Method	No. of Patients	Age of Patients (Mean)	Dose	Outcomes	Cytotoxic Effects	Additional Notes	Reference
2010	Autologous DCs	ALL	WT1 mRNA electroporation	10	31–83 (61)	4 shots 2 weeks interval 5, 10, or 20 × 10^6^	2CR	–		[92]
2013	Autologous DCs	CD38+ and CD138+ MM	Fusion with whole MM cell	100	35–70	3–4 shots 2 × 10^6^/kg per shot	78% R 47% VGPR 31% CR	–	57 % two-year progression-free survival	[93]
2018	Allogenic DCs	AML	Differentiated from AML cell line	12	57–74 (68)	4 shots 2 weeks interval 10, 25 or 50 × 10^6^	5 CR 4 PD	–	Post-remission treatment	[94]
2013	Autologous T cells	CD19+ ALL	2nd gen CAR (+CD28)	16	23–74 (50)	One shot 1.5–3 × 10^6^/kg	14 OR 10 CR 13 MRD-	2 CRS4 2 CRS3		[17]
2014	Autologous T cells	CD19+ B-ALL and T-ALL	2nd gen CAR (+4-1BB)	30	5–60	One shot 0.76–20 × 10^6^/kg	27 CR 19 SCR	Severe in 8 patients	FDA approved for ALL and DLBCL Produced by Novartis under the commercial name Kymriah	[25]
2016	Autologous T cells	CD20+ B-cell NHL	2nd gen CAR (+CD137ζ)	11	25–70 (59)	One shot 0.5–1.5 × 10^6^/kg	6 CR 3 PR 2 SD	2 CRS3		[28]
2017	Autologous T cells	CD19+ B-cell lymphomas	2nd gen CAR (+CD28)	111	23–76	One shot 2 × 10^6^/kg	82% OR 54% CR	95% CRS3+	FDA-approved for B-cell lymphoma Produced by Gilead under the commercial name Yescarta	[95]
2017	Autologous T cells	CD22+ lymphoma and leukemia	2nd gen CAR	21	7–30 (19)	One shot 0.3–3 × 10^6^/kg	12 CR 9 MRD-	1 CRS4 1 CRS3	17 patients were resistant to CAR anti-CD19 in the past	[29]
2019	Autologous T cells	CD19+ and BCMA+ MM	2nd gen CAR	21	49–61 (58)	One shot 1 × 10^6^ + 1 × 10^6^/kg	12 CR 8 PR 1 SD 17 MRD-	1CRS3 2 NT		[96]
2018	NK92 cell line	CD33+ AML	3rd gen CAR (+CD28 and 4-1BB)	3	14–49	Three shots 2 days interval 300, 600, 1000 × 10^6^	No response	2CRS1	One patient died of GvHD after chemotherapy and donor lymphocyte infusion	[65]
2020	Allogenic NK cells from cord blood	CD19+ lymphoma and leukemia	2nd gen CAR (+CD28)	11	23–66 (52)	One shot 0.1, 1 or 10 × 10^6^/kg	8 OR 7 CR	No GvHD No CRS	No GvHD despite some HLA mismatch; Toxic events related to lymphodepletion	[66]

Abbreviations: OR, objective response. CR, complete response. VGPR, very good partial remission. PR, partial response. SD, stable disease. PD, progressive disease. MRD, minimal residual disease negative. SCR, sustained complete response. CRS, cytokine release syndrome (+grade). NT, neurotoxicity. DCs, dendritic cells. MM, multiple myeloma. CARs, chimeric antigen receptors. AML, acute myeloid leukemia. BCMA, B-cell maturation antigen. NK, natural killer. GvHD, graft-versus-host-disease. HLA, human leukocyte antigen. NHL, non-Hodgkin’s lymphoma. ALL, acute lymphoblastic leukemia. DLBCL, diffuse large B-cell lymphoma.

**Table 2:** Selected cell-based clinical trials and preclinical studies with stem cells to treat hematological malignancies, featuring key aspects. iPSCs: induced pluripotent stem cells, HSCs: hematopoietic stem cells, MSCs: mesenchymal stem cells, GvT: graft versus tumor effect, TRAIL: TNF-related apoptosis-inducing ligand, and NKT: natural killer T cells.

Cell Source	Target	Mode of Action	Outcomes	Reference
HSCs from cord blood	Patients with AML or ALL	GvT	Decrease in leukemic relapse	[107]
MSCs from bone marrow	Mouse models with leukemia cell lines MOLT-4 and L1210	Loaded with paclitaxel	Antileukemia activity in vitro and in the mouse model	[108]
MSCs from adipose tissue	MM cell lines in vitro: RPMI-8226 U-266, MMCAR-1, LIG-1, and MCC-2	Engineered with the expression of TRAIL	Antimyeloma activity in vitro	[109]
iPSCs from murine embryonic fibroblasts	Murine T-cell lymphoma	Differentiation to NKT cells	Tumor growth suppression in the mouse model	[110]
iPSCs from murine embryonic fibroblasts	Raji human Burkitt lymphoma cell line	Differentiation to T cells expressing CD19 CAR	Tumor growth inhibition in the mouse model	[111]

## Abbreviations

ALL	Acute lymphoblastic leukemia
AML	Acute myeloid leukemia
AP-1	Activator protein 1
B16	Name of murine melanoma cell line
BCL	B cell lymphoma
BCMA	B-cell maturation antigen
CAR-T	Chimeric antigen receptor T-cell
CARs	Chimeric antigen receptors
Cas9	CRISPR associated protein 9
CCL25	C-C motif chemokine ligand 25
CCR2	C-C motif chemokine receptor 2; cluster of differentiation 192
CCR9	C-C motif chemokine receptor 9
CD137 ζ	Cluster of differentiation 137 ζ
CD138	Syndecan-1; Cluster of differentiation 138
CD14	Cluster of differentiation 14
CD16	Cluster of differentiation 16
CD19	Cluster of differentiation 19
CD20	Cluster of differentiation 20
CD22	Cluster of differentiation 22
CD244	Cluster of differentiation 244
CD28	Cluster of differentiation 28
CD3	Cluster of differentiation 3
CD30	Tumor necrosis factor receptor superfamily member 8; Cluster of differentiation 30
CD33	Cluster of differentiation
CD3ζ	Cluster of differentiation 3ζ
CD4	Cluster of differentiation 4
CD8	Cluster of differentiation 8
CLL	Chronic lymphocytic leukemia
CML	Chronic myelogenous leukemia
CR	Complete response
CRISPR	Clustered regularly interspaced palindromic repeats
CRS	Cytokine release syndrome
DCs	Dendritic cells
DLBCL	Diffuse large B-cell lymphoma
EG7	Name of cell line derived from murine T-cell lymphoma
EGFRt	Epidermal growth factor receptor
EPCs	Endothelial progenitor cells
FcRγ	Fc receptor gamma
FDA	Food and Drug Administration
GvHD	Graft versus host disease
GvT	Graft versus tumor effect
HLA	Human leukocyte antigen
HSCs	Hematopoietic stem cells
IFN-γ	Human interferon gamma
IL-2	Interleukin 2
iPS-MEF-Ng-20D-17	Name of mouse induced pluripotent stem cell line
iPSCs	Induced pluripotent stem cells
K562	Human erytroleukemic cell line
Klf4	Kruppel-like factor 4
L1210	Name of murine cancer cell line derived from lymphoblasts
LeY	Lewis Y antigen
M1	Macrophage phenotype 1
M2	Macrophage phenotype 2
MDS	Myelodysplastic syndrome
Megf10	Multiple epidermal growth factor-like domains 10
MHC	Major histocompatibility complex
MM	Multiple myeloma
MRD	Minimal residual disease
mRNA	Messenger ribonucleic acid
MSCs	Mesenchymal stem cells
Myc	Master regulator of cell cycle entry and proliferative metabolism
Nalm6	Human B cell precursor leukemia cell line
NF-κB	Nuclear factor kappa-light-chain-enhancer of activated B cells
NFAT	Nuclear factor of activated T-cells
NHL	Non-Hodgkin’s lymphoma
NK	Natural killer
NK-92	Name of human NK cell line
NK1.1	NK cell-associated marker 1.1; cluster of differentiation 161
NKG2D	Natural killer group 2D receptor
NKp46	Natural cytotoxicity triggering receptor 1; cluster of differentiation 335
NKT	Natural killer T cells
NT	Neurotoxicity
NY-ESO-1	Cancer-testis antigen
Oct3/4	Octamer-binding transcription factor 4
OP9	Name of murine embryonic cell line
OR	Objective response
PD	Progressive disease
PDGF-BB	Platelet-derived growth factor BB
PR	Partial response
RD114	Name of envelope glycoprotein of lentiviral vectors
RPMI8226	Name of multiple myeloma human cancer cell line derived from B lymphocytes
scFv	Single-chain variable fragment
SCR	Sustained complete response
SCs	Stem cells
SD	Stable disease
SDF-1	Stromal cell derived factor 1
Sox2	Sex determining region Y-box 2
sTRAIL	Soluble TNF-related apoptosis-inducing ligand
SUPRA	Split Universal Programmable
TAAs	Tumor-associated antigens
TCR	T cell receptor
TGF-**β**	Transforming growth factor **β**
TNF-α	Tumor necrosis factor alpha
TRAIL	Tumor necrosis factor-related apoptosis-inducing ligand
TRUCKs	T cells redirected for universal cytokine-mediated killing
U-266	Name of human cancer cell line derived from B lymphocyte
VEGF	Vascular endothelial growth factor
VGPR	Very good partial remission
VSV-G	Vesicular stomatitis virus G
WT1	Wilms’ tumor 1
4T1	Murine mammary tumor cell line
